# A Case Report of Cake Frosting as a Source of Copper Toxicity in a Pediatric Patient

**DOI:** 10.5811/cpcem.2020.7.47267

**Published:** 2020-08-02

**Authors:** Hoi See Tsao, Lauren Allister, Takuyo Chiba, Jonathan Barkley, Rose H. Goldman

**Affiliations:** * Warren Alpert Medical School of Brown University, Department of Emergency Medicine, Providence, Rhode Island; † Warren Alpert Medical School of Brown University, Department of Pediatrics, Providence, Rhode Island; ‡ Brown University School of Public Health, Providence, Rhode Island; § Harvard Medical School, Boston Children’s Hospital and the Beth Israel Deaconess Medical Center, Department of Toxicology, Boston, Massachusetts; ¶ Rhode Island Department of Health, Center for Acute Infectious Disease Epidemiology, Providence, Rhode Island; || Harvard Medical School, Department of Medicine, Boston, Massachusetts; ** Harvard T.H. Chan School of Public Health, Department of Environmental Health, Boston, Massachusetts; †† Cambridge Health Alliance, Division of Occupational and Environmental Medicine, Cambridge, Massachusetts; ‡‡ New England Pediatric Environmental Health Specialty Unit, Boston, Massachusetts

**Keywords:** copper, toxicology, ingestion, poison, pediatric

## Abstract

**Introduction:**

Copper is an uncommon source of metal toxicity in children that requires a high index of suspicion for diagnosis.

**Case Report:**

We describe the unique presentation of a 12-month-old girl who developed acute onset of vomiting and diarrhea after ingestion of a copper-contaminated birthday cake.

**Conclusion:**

This case highlights the presentation, evaluation, and management of the rare pediatric patient who presents with copper poisoning. This case also illuminates the public health implications of potential metal poisoning when using non-edible decorative products in homemade and commercially prepared baked goods.

## INTRODUCTION

Copper is an essential trace element in humans that is used as a cofactor in many redox reactions, including mitochondrial oxidative phosphorylation, free radical detoxification, neurotransmitter formation, pigment synthesis, connective tissue synthesis, and iron metabolism. Low amounts of copper are found in foods such as animal liver, crustaceans, shellfish, green vegetables, dried fruit, nuts, and chocolate. High levels of copper can cause toxicity, often secondary to exposure to pesticides, fungicides, copper-contaminated pipe water and water treatment systems.^1^-^2^ We present a review of the literature on copper poisoning and a case report of a pediatric patient presenting to the emergency department (ED) following a known copper ingestion.

## CASE REPORT

**Image 1:**
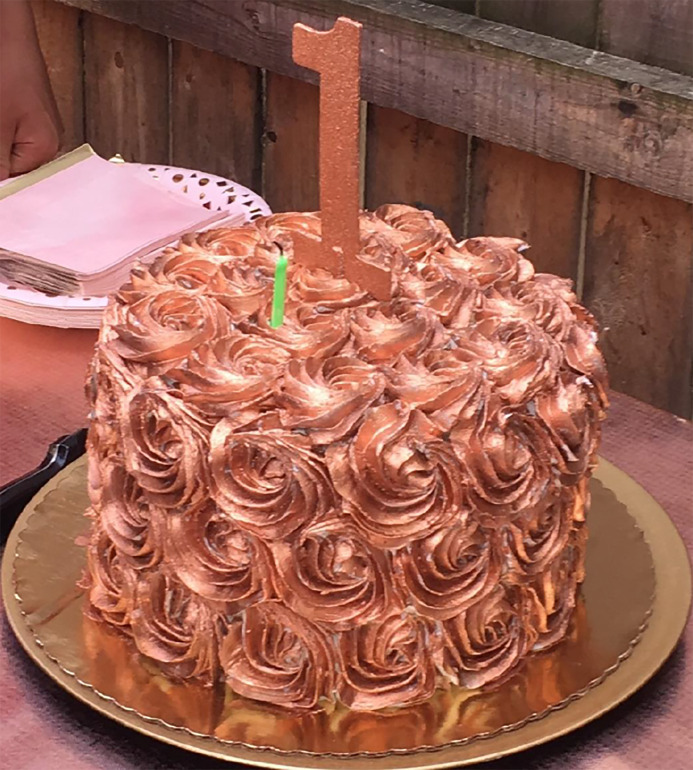
Birthday cake with rose-gold luster dust frosting, the consumption of which led to copper toxicity.

**Image 2:**
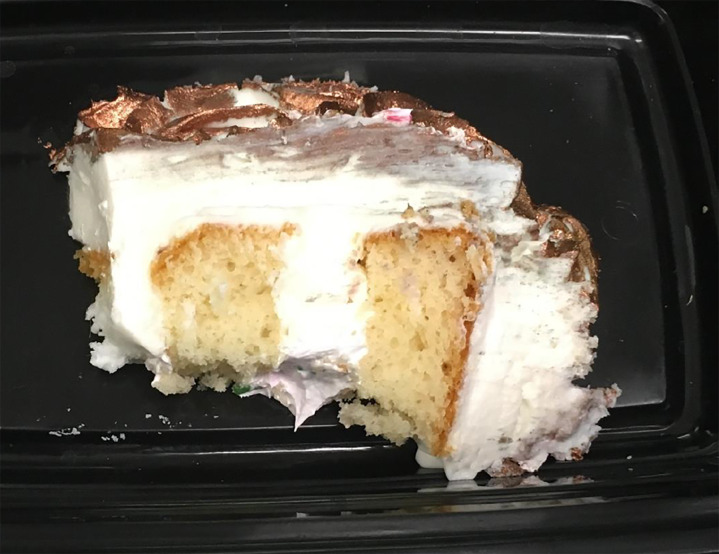
Slice of birthday cake with rose-gold luster dust frosting, the consumption of which led to copper toxicity.

A 12-month-old female with no significant medical history presented to the ED with listlessness four days following one day of resolved gastrointestinal symptoms. Her symptoms began after ingestion of birthday cake with rose-gold frosting from a local bakery (Images 1 and 2).

Within 20 minutes of cake consumption, the patient experienced six episodes of non-bloody, non-bilious vomiting and several episodes of non-bloody diarrhea. Multiple other guests developed similar symptoms that resolved after several hours.

**Image 3:**
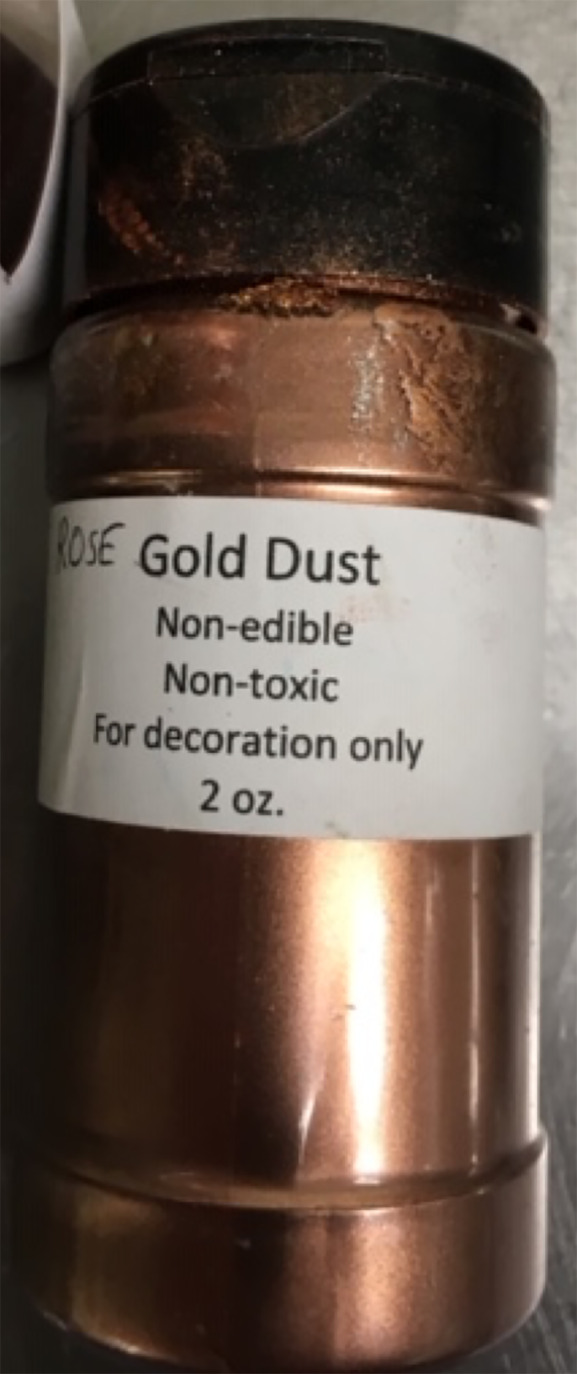
Rose-gold luster dust vial used to provide decorative color to cake frosting, resulting in copper toxicity.

The patient saw her pediatrician, who suspected a foodborne illness or other toxic exposure and called the regional poison control center, which in turn involved the Department of Health (DOH). The DOH found that only guests who ate frosted cake developed vomiting or diarrhea. Guests who did not eat the cake or ate cake without frosting had no symptoms. The DOH did not report the discovery of bacteria or other infectious agents. The DOH conducted an investigation into the bakery. The cake had been frosted with a rose-gold luster dust labeled “non-edible non-toxic for decoration only” (Image 3) that was mixed into a butter extract and painted onto the cake.

Based on the material safety data sheet provided by the supplier, the luster dust contained elemental copper. Chemical testing of the luster dust and leftover frosted birthday cake was performed by the state health laboratory. The cake frosting contained 21.1 milligrams (mg) of copper per gram. Each cake slice was estimated to contain 40 grams of frosting. Thus, each slice contained approximately 900 mg of copper.^4^ For comparison, beef liver, a copper-rich food, contains 0.157 mg of copper per gram, or 17 mg of copper for a 4-ounce portion.^5^ The DOH reported that the symptoms, timeline of illness, and laboratory evidence were consistent with copper poisoning from cake frosting.^4^

The investigation results were released four days after the patient’s exposure to the copper-contaminated cake. On the same day, she developed listlessness, poor oral intake, and what the parents thought was a facial droop. The pediatrician was concerned these symptoms could be due to ongoing copper toxicity. After consultation with the regional poison control center, the patient was referred to the ED.

**CPC-EM Capsule**

What do we already know about this clinical entity?

Copper poisoning is an uncommon metal toxicity that is often secondary to exposure to pesticides, fungicides, contaminated pipe water or water treatment systems.

What makes this presentation of disease reportable?

We describe the presentation, evaluation, and management of a pediatric patient with copper poisoning from a previously unreported source.

What is the major learning point?

Popular decorative products used in the commercial baking industry are a potential source of copper or other heavy metal poisoning.

How might this improve emergency medicine practice?

This newly recognized source of copper toxicity requires a high index of clinical suspicion from providers to ensure appropriate history-taking, evaluation, and management.

The patient’s ED vital signs were temporal temperature 36.9 degrees Celsius; heart rate 130 beats per minute; respiratory rate 40 breaths per minute; blood pressure 93/77 millimeters of mercury, and oxygen saturation 95% on room air. Physical exam showed a happy, interactive child with normal pulmonary, cardiac, abdominal, neurologic (no facial droop or focal neurologic symptoms), and skin examinations. The regional poison control center was contacted to discuss management given the concern for copper toxicity. A comprehensive metabolic panel and complete blood count were normal. A nasal swab was positive for rhinovirus/enterovirus. Copper and ceruloplasmin levels were sent. Given the patient’s well appearance, normal vital signs, normal laboratory results, and the unlikely possibility of copper-related delayed neurologic effects, no chelation was started pending copper studies. The patient was admitted to the hospital for observation.

On hospital day two, the patient’s total serum copper level resulted at 97 micrograms per deciliter (mcg/dL) (reference range 85–185 mcg/dL) and her ceruloplasmin level was 22 mg/dL (reference range 20–60 mg/dL). Her calculated non-ceruloplasmin bound or free copper level was elevated at 31 mcg/dL (reference range 0–10 mcg/dL). Her physical exam remained normal. No chelation therapy or other interventions were initiated, and she was discharged home.

One month later, she was seen in the pediatric environmental health clinic. Her parents reported that the patient was back at her baseline. She had a normal physical examination and normal laboratory tests including total serum copper of 94 mcg/dL (reference range 70–150 mcg/dL) and free non-ceruloplasmin-bound copper <2.5 mcg/dL (reference range 0–10 mcg/dL).

The bakery was fined and prohibited from using the rose-gold luster dust and any other decoration unless specifically labeled as edible. The DOH visited additional establishments and issued guidance about these products to all bakeries in the state.

## DISCUSSION

The mechanism of action of copper toxicity is through the creation of reactive oxygen species that cause oxidative cell damage and death.^2^ Copper ingestion typically presents first with gastrointestinal symptoms including vomiting and abdominal pain, followed by gastroduodenal hemorrhage, ulceration or perforation in severe cases. Copper is then bound rapidly from the gastrointestinal tract by carrier proteins, ceruloplasmin and albumin, and transported to the liver and other tissues, where it can cause hepatotoxicity, methemoglobinemia and rhabdomyolysis. Hemolysis can occur within 24 hours from ingestion.^1^,^6^,^7^ The biological half-life of copper ranges from 13–33 days,^8^ and is predominantly eliminated via biliary excretion at an average rate of 2 mg per 24 hours.^7^

Prior studies on copper toxicity have focused on copper salt ingestions, such as copper sulfate. Elemental copper ingestions, such as in coin ingestions, usually do not cause toxicity unless in an acidic environment when elemental copper can transform into reactive copper ions. There have been prior copper toxicity cases in the setting of consuming beverages exposed to copper-contaminated bottle pourers, boilers, and cocktail shakers.^9^ The State of Iowa Alcoholic Beverages Division therefore recommends avoiding using unlined copper mugs for beverages with a pH below 6.0, such as Moscow Mules.^3^ There are no published cases of copper powder ingestion causing toxicity. This patient case is therefore the first documented case of elemental copper powder ingestion causing toxicity in humans.

This case highlights the challenges in diagnosing and treating copper toxicity. If the patient had presented to the ED prior to the DOH investigation, given the simultaneous onset of gastrointestinal symptoms among the other guests, a foodborne microbial or toxin-mediated etiology may have been suspected instead of metal poisoning. However, it is important for clinicians to note that the onset of nausea and vomiting within 30 minutes of exposure is more consistent with metal or toxicant poisoning in contrast to foodborne illness, which typically takes several hours from exposure to symptom development. The patient’s listlessness and poor oral intake during her ED visit four days after cake ingestion were also likely due to rhinovirus, as diagnosed by nasal swab, rather than ongoing copper toxicity.

Laboratory testing for copper toxicity, to confirm diagnosis or direct treatment, presents several challenges. Serum copper levels are not associated with copper toxicity severity.^6^ Additionally, no standardized serum copper reference range exists for infants, toddlers, or young school-age children. The youngest children for which a reference range is available are 6- to 11-year-old children, where the average serum copper concentration was 119 mcg/dL, and 95^th^ percentile was 157 mcg/dL.^10^ We identified only one article in the literature that evaluated copper concentrations among healthy 6-month-old to 2-year-old children and showed an average serum copper concentration of 111 mcg/dL (standard deviation 26, range 72–178).^11^

The lack of a standardized reference range for infants was reinforced when the two hospitals that tested the patient’s serum copper level provided different reference ranges: 85–185 mcg/dL (Lifespan hospitals, all ages); and 70–150 mcg/dL (Boston Children’s Hospital, with a note that mean levels are higher in women and children). While there is limited data on standardized serum copper level reference ranges for infants, our patient’s serum copper level of 97 mcg/dL was within a similar normal reference range from available studies. However, the time from her exposure to copper level blood draw was four days, which may have allowed for normalization of her serum copper concentration.

Finally, even in the setting of normal total serum copper and ceruloplasmin levels, non-ceruloplasmin-bound or free copper levels can be elevated, which can suggest excessive copper ingestion. Based on Wilson’s disease studies, routine serum copper levels performed in laboratories include both bound and unbound serum copper and may be falsely normal depending on ceruloplasmin levels.^12^ To determine the form of copper that is free for deposition in tissues and toxic to cells, the non-ceruloplasmin-bound copper level should be calculated. The non-ceruloplasmin-bound copper level is calculated using the following equation: Non-ceruloplasmin-bound copper (mcg/dL) = serum copper (mcg/dL) – (3 × serum ceruloplasmin [mg/dL]).^12^ In our patient, the calculated non-ceruloplasmin-bound copper level was elevated at 31 mcg/dL (reference range 0–10 mcg/dL).

The management of acute copper poisoning includes supportive care, fluid resuscitation for hemodynamic instability, replacement of gastrointestinal losses, and symptom management. Useful lab studies include those that evaluate for end-organ damage from copper deposition, dehydration, rhabdomyolysis, and methemoglobinemia in cyanotic patients. The interpretation of copper studies, especially in pediatric patients, can be difficult to reconcile with clinical symptoms. Normal values do not exclude exposure. The timing and type of copper biomonitoring are crucial given copper metabolism and non-standardized copper reference ranges. Regional poison control centers should be consulted to guide evaluation and management, including the decision for chelation. While there are limited studies regarding the efficacy of chelation, it should be considered when there are hepatic, hematologic, or other severe manifestations of toxicity.^12^ Clinically available chelators include oral penicillamine, oral triethylenetetramine (Trientine), and intramuscular dimercaprol.^7^ If considering chelation, poison control center consultation is recommended to formulate individualized management plans, especially to engage in risk-benefit decision-making regarding chelation.

From a public health perspective, it is important for home and commercial bakers to be aware of the dangers of metal poisoning from decorative products. The DOH visited additional bakeries and found that one-third of the bakeries were using inedible luster dust on edible food products. The DOH issued guidance to bakeries, clarifying that the label “non-toxic” does not equate to being edible, and that edible luster dusts must have an ingredient list on the product label. The public health investigation into this particular case initiated other state investigations with similar findings of metals, such as lead, in decorative cakes.^13^ The United States Food and Drug Administration has since released guidelines on how to use these decorative products appropriately and ways to determine whether decorative products are safe and edible.^14^

## CONCLUSION

Copper poisoning in children is rare and may be difficult to diagnose but can have significant morbidities.^1^,^2^ When copper poisoning is suspected, laboratory studies that evaluate for end-organ damage, dehydration, rhabdomyolysis, and methemoglobinemia should be obtained. The utility of copper studies is limited. Treatment consists of predominantly supportive care, and the decision for chelation should be made in consultation with the regional poison control center. A new source of this uncommon metal poisoning is decorative products used in popular custom-made specialty baked goods, and requires a high index of clinical suspicion to ensure appropriate history-taking, evaluation, and management.
